# The lateral habenula regulates stress-related respiratory responses via the monoaminergic system

**DOI:** 10.1007/s00424-024-03043-7

**Published:** 2024-11-19

**Authors:** Riko Mizukami, Masayuki Matsumoto, Tadachika Koganezawa

**Affiliations:** 1 Department of Neurophysiology, Division of Biomedical Science, Institute of Medicine, University of Tsukuba, Tsukuba, Ibaraki 305-8575 Japan; 2 Doctoral Program in Neuroscience, Graduate School of Comprehensive Human Sciences, University of Tsukuba, Tsukuba, Ibaraki 305-8575 Japan; 3 Department of Cognitive and Behavioral Neuroscience, Division of Biomedical Science, Institute of Medicine, University of Tsukuba, Tsukuba, Ibaraki 305-8575 Japan; 4 Center for the Evolutionary Origins of Human Behavior, Kyoto University, Inuyama, Aichi 484-8506 Japan

**Keywords:** Dopamine, Lateral habenula, Respiration, Stress, Ventral tegmental area

## Abstract

Psychologic stress induces behavioral and autonomic responses such as acceleration of respiration. The lateral habenula (LHb) is noted to be involved in stress-induced behavioral responses. However, its involvement in stress-induced respiratory responses is unknown. In this study, we aimed to analyze whether and how the LHb regulates respiration. Electrical stimulation of the LHb of anesthetized Wistar male rats increased respiratory frequency and minute ventilation, calculated by respiratory frequency × thoracic movement amplitude. Systemic administration of a dopaminergic receptor antagonist, clozapine, suppressed the LHb-induced respiratory responses. On the other hand, administration of a serotonergic receptor antagonist, methysergide, significantly accelerated the LHb-induced increase in respiratory frequency, together with suppressing the thoracic movement amplitude. To clarify the source of dopaminergic modulation, we inhibited the ventral tegmental area (VTA), which contains dopaminergic neurons and receives inputs from the LHb, by administering microinjections of a GABA_A_ agonist, muscimol. The bilateral inhibition of the VTA almost abolished the LHb-induced respiratory responses. These results suggest that LHb activation causes respiration acceleration, mainly mediated by dopaminergic neurons in the VTA and suppressively modulated by the serotonergic system. Neural circuits originating in the LHb may be a key modulator for respiration during psychological stress.

**Supplementary Information:**

The online version contains supplementary material available at 10.1007/s00424-024-03043-7.

## Introduction

Stress stimuli cause a specific response in respiration. This stress-induced respiratory response is characterized by increased ventilatory output [4, 6, 7, 9, 16, 22, 26, 32]. The respiratory change is usually helpful in supporting behavioral reactions during stress events. However, the respiratory responses sometimes cause a sense of dyspnea due to hyperventilation in stress-triggered disorders such as hyperventilation syndrome and panic attacks. Despite this, the neural basis underlying the respiratory response triggered by stress remains unclear.

The lateral habenula (LHb) is located in the dorsal part of the diencephalon, receives inputs from the forebrain limbic structures, and sends projections to the monoaminergic system [23, 25, 38, 50]. The LHb is known to participate in stress-induced behavior and learning [30, 31, 49], and overactivation of the LHb was observed in patients with major depressive disorder [29]. Also, deep brain stimulation treatment to the LHb improves the symptoms of depression [2, 40]. Therefore, the LHb is thought to be a center of stress-induced behavioral responses. Moreover, the neurons in the LHb are activated by stress stimuli and regulate stress-induced responses mediated by the monoaminergic system [1, 34]. Dopamine is one of the best-known neurotransmitters in the brain. The dopaminergic system plays essential roles in response to conditioned fear and pain and is also involved in the respiratory system [3, 5, 11, 15, 17–20, 39, 42]. In addition, the serotonergic system is known to be responsible for the respiratory circuits [12, 27, 36]. LHb activation also induces stress-induced cardiovascular responses via the serotonergic system [13]. Whilst it has been known that respiratory movement is modulated by some neuromodulators, such as monoamine with acetylcholine, histamine, ATP, substance P, and cholecystokinin [14], whether the LHb modulates respiration with the monoaminergic system is unclear.

In this study, we hypothesized that the LHb regulates the stress-induced respiratory response via the midbrain monoaminergic system. To examine this hypothesis, we applied electrical stimulation of the LHb in anesthetized Wistar male rats and observed the effects on respiratory movement. Here, we show that LHb activation causes acceleration of respiration and that LHb-induced respiratory responses are suppressed by blockade of dopaminergic receptors and inactivation of the ventral tegmental area (VTA), a major dopamine source. This finding suggests that the LHb regulates respiratory responses via the dopaminergic system, especially from the VTA.

## Methods

### Animal preparation

Eleven-week-old male Wistar rats (Japan SLC, Inc.) were kept under standard laboratory conditions consisting of a 12-h light period and 12-h dark period and maintained at 25 °C with free access to food and water. The rats weighed 300 to 450 g. They were first anesthetized with isoflurane (Fujifilm Wako Pure Chemical Corporation) and then maintained under anesthesia with urethane (1–1.25 g/kg body weight, *i.p*.; Tokyo Chemical Industry Co., Ltd.). Anesthesia was confirmed by the loss of response to hindlimb pinch stimulation. A heparin (200 IU/mL)-filled catheter (SP-28; Natsume Seisakusyo Co., Ltd.) was inserted into the femoral artery and connected to a carrier amplifier (AP-621G; Nihon Kohden) for blood pressure measurement. The heart rate was measured by use of electrocardiography and amplified by use of a bioelectrical amplifier (AP-651 J; Nihon Kohden). A catheter (SP-28, Natsume Seisakusyo Co., Ltd.) filled with saline was inserted into the femoral vein for intravenous administration. The rats were fixed in a stereotaxic apparatus. The body temperature was kept warm at around 36 °C with a heating pad (BWT-100A; Bio Research Center Co., Ltd).

### Measurement of respiratory movement

An isotonic transducer (TD-11, Nihon Kohden) was attached to the neck of the rat (5 cm caudally from the bregma) to observe respiratory movement. The vertical thoracic movement produced by respiration was measured by the transducer and recorded as a waveform. Respiratory frequency and thoracic movement amplitude were calculated from the interpeak intervals and the peak-to-peak respiratory movements, respectively. Since thoracic movement amplitude is correlated with tidal volume [45, 52], the product of respiratory frequency and thoracic movement amplitude was used as the value reflecting minute ventilation.

### Electrical stimulation of the LHb

**Fig. 1:**
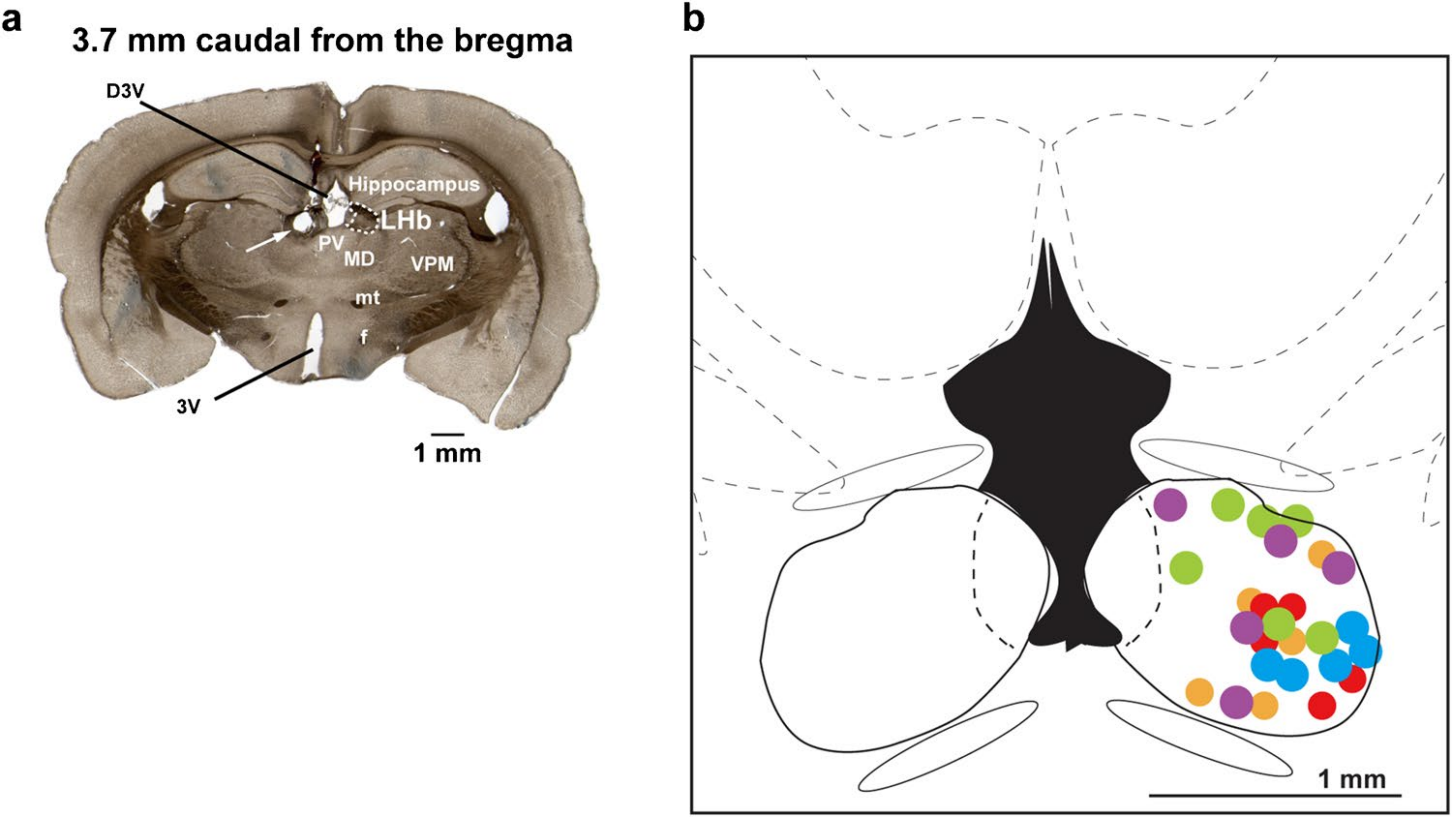
Histologic examination. **a** Histologic examination of the stimulation site. The slice is taken from 3.7 mm caudally from the bregma, which corresponds to 3.6 mm caudally from the bregma on the Paxinos and Watson rat brain atlas [21]. The arrow indicates a lesioned site that indicates a stimulation site in the LHb. The area enclosed by the dotted line is the LHb. D3V: Dorsal 3rd ventricle, f: fornix, LHb: lateral habenula, MD: Mediodorsal thalamic nucleus, mt: mammillothalamic tract, PV: Palaventricular thalamic nucleus, VPM: Ventral posteromedial thalamic nucleus, 3V: 3rd ventricle. **b** The orange dots show the stimulation sites in the experiments used to observe the effects of LHb stimulation on respiratory movement (*n* = 5). The red, blue, and purple dots show the stimulation sites in the LHb with the administration of clozapine (*n* = 5), the vehicle (*n* = 5), and methysergide (*n*= 5). The green dots show the stimulation sites in the LHb with the administration of muscimol to the VTA (*n* = 6)

A coaxial electrode (200 μm tip diameter, Unique Medical Co., Ltd.) was placed into the left LHb for electrical stimulation. The stimulated sites were located 3.2 to 3.6 mm caudally from the bregma (which corresponds to 3.14 to 3.6 mm caudally from the bregma on the Paxinos and Watson rat brain atlas [21]), 0.4 to 1.2 mm laterally from the midline, and 3.1 to 5 mm ventrally from the cortical surface in the LHb. The intensity of electrical stimulation was 50 μA to 1 mA; the frequency, 10 to 100 Hz; and the stimulation interval, 0.5 ms for 10 s. Each stimulus was performed with a minimum interval of 200 s. At the end of the experiment, a lesion was made by means of a direct current (100 μA for 10 s) through the stimulation electrode. The lesion was used to confirm the stimulated position histologically (Fig. 1).

### Administrations of dopamine and serotonin receptor antagonists

To determine the involvement of the monoaminergic system in LHb-induced respiratory responses, we administered a dopamine receptor antagonist, clozapine (1 mg/kg, *i.v.*; Fujifilm Wako Pure Chemical Corporation) [43], and a serotonin receptor antagonist, methysergide maleate (1 mg/kg, *i.v.*; Abcam) [46], via the femoral vein. At first, 10 mg clozapine was dissolved in 0.1 mol hydrochloric acid (1 mL), and then clozapine was diluted with saline to the desired concentration (1 mg/mL). Methysergide was dissolved in saline.

To examine if the VTA mediates LHb-induced respiratory responses, we pharmacologically inactivated the VTA. A GABA_A_ receptor agonist, muscimol (10 mM in saline with 2% pontamine sky blue, 100 nL; Abcam), was locally injected into the bilateral VTA with a microsyringe attached to the stereotaxic apparatus. The centers of the injected sites were located 4.3 to 5.1 mm caudally from the bregma (which corresponds to 4.8 to 5.3 mm caudally from the bregma on the Paxinos and Watson rat brain atlas [21]), 0.6 to 1.8 mm laterally from the midline, and 8.0 to 9.0 mm ventrally from the cortical surface in the VTA.

### Histologic examination

At the end of the experiment, perfusion was performed with 200 mL of saline from the left ventricle, which was followed by fixation with 200 mL of 10% formalin (Fujifilm Wako Pure Chemical Corporation). The brain was removed and submerged in 10% formalin and maintained at 4 °C for at least 24 h. After that, 50-µm-thick frozen slices were prepared by use of a microtome with cooling capability. Each slice was scanned with a histology slide scanner (primehisto XE, PacificImage Electronics) to identify the stimulated and drug-administered sites.

### Data analysis

All the data were digitized by use of an AD converter (1401 plus, Cambridge Electronic Design Limited) and processed and analyzed by use of analysis software (Spike 2, Cambridge Electronic Design Limited). Excel (Microsoft) and IBM SPSS Statistics (IBM) were also used for the analysis.

To calculate the change rates in respiratory frequency, thoracic movement amplitude, and (thoracic movement amplitude) × (respiratory frequency) by the LHb stimulation, the average of 100 s before the LHb stimulation was determined as 100%. The average value of 5–10 s from the onset of LHb stimulation was expressed as the percent change. In the pharmacologic experiments, changes in respiratory responses to stimulation of the LHb before and after drug administration were examined to determine the effect of the administration.

### Statistics

Numeric data were expressed as mean ± SEMs. To compare 2 different groups, we used the paired *t*-test. To compare multiple groups, we used ANOVA with repeated measurements followed by the Dunnett test for comparison with the control. Significance levels were set at *p* < 0.05.

## Results

### Effects of LHb stimulation on respiratory movement

**Fig. 2:**
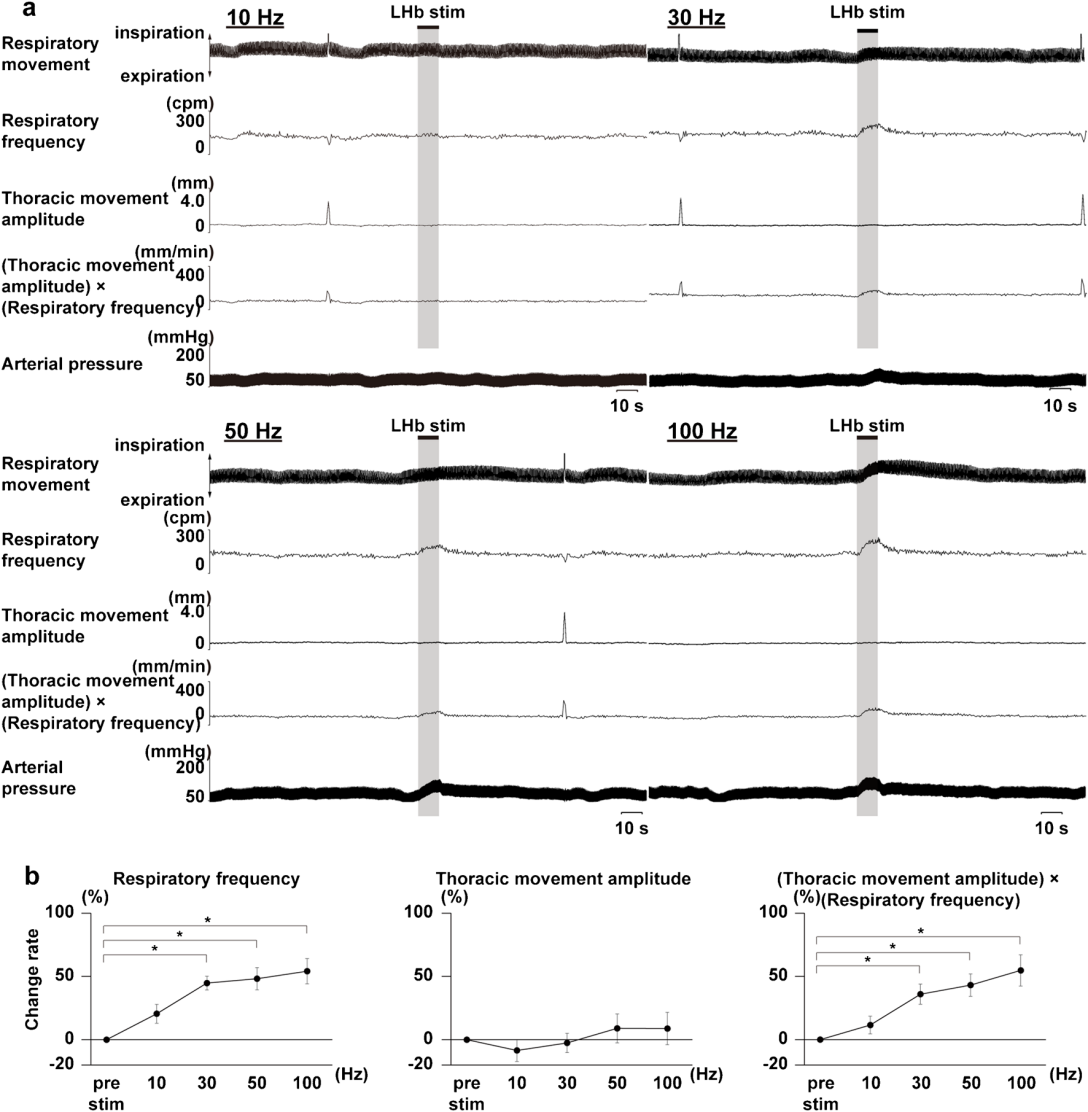
LHb stimulus frequency-dependent changes in respiratory responses. **a** Respiratory responses elicited by LHb stimulation at each stimulus frequency (10 Hz, 30 Hz, 50 Hz, and 100 Hz). The black bars at the top show the period of LHb stimulation (10 s). LHb: lateral habenula. cpm: cycles per minute. **b** Analyzed respiratory responses during LHb stimulation at each stimulus frequency. Asterisks indicate *p* < 0.05 vs prestimulus (*n* = 5). cpm: cycles per minute, bpm: beats per minute

**Fig. 3:**
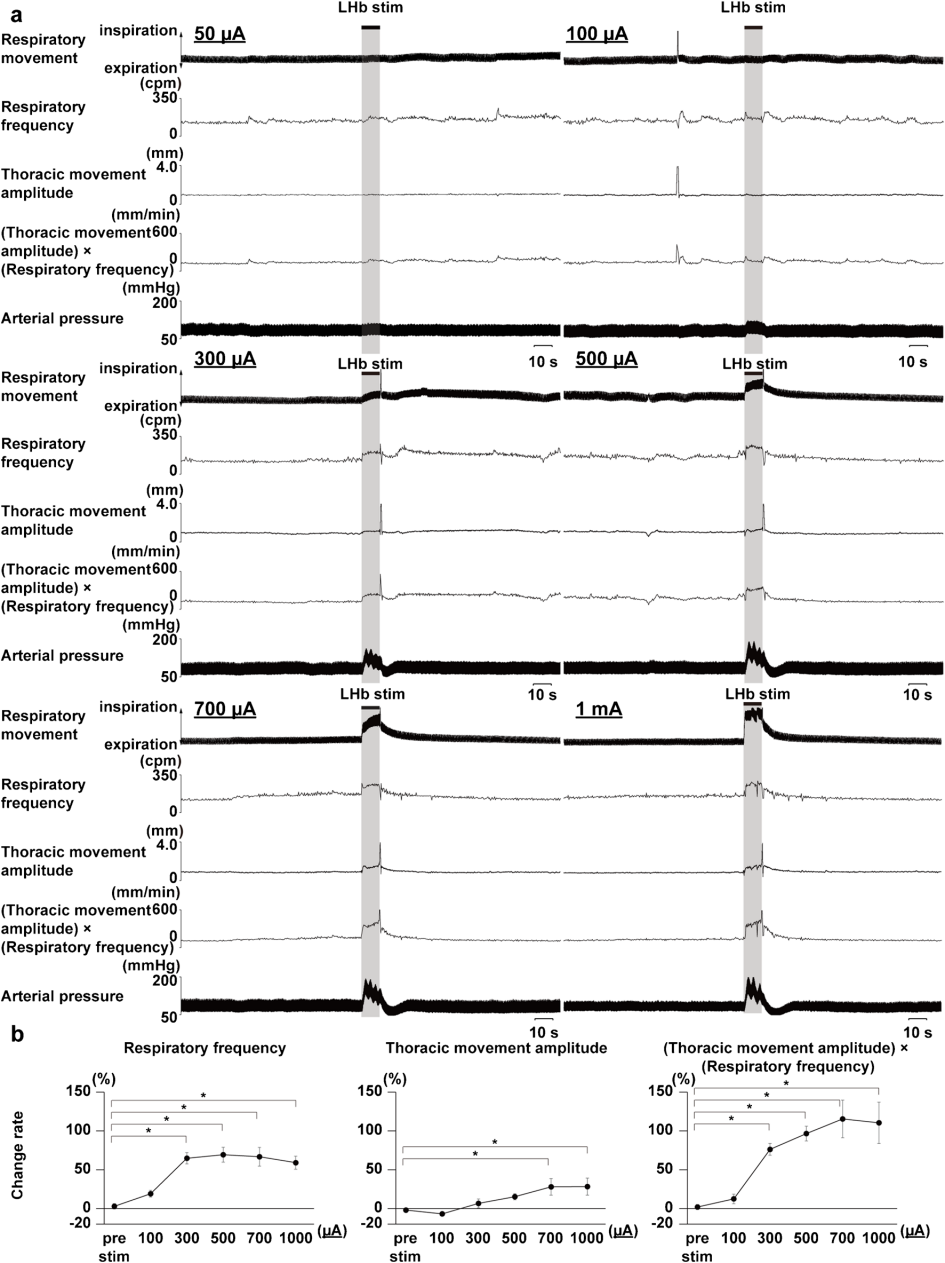
Stimulus intensity-dependent changes in respiratory responses elicited by LHb stimulation. **a** Respiratory responses elicited by each stimulus intensity (50 µA, 100 µA, 300 µA, 500 µA, 700 µA, 1 mA). The black bars at the top show the period of LHb stimulation (10 s). LHb: lateral habenula. cpm: cycles per minute. **b** Analyzed respiratory responses during LHb stimulation at each stimulus intensity. Asterisks indicate *p* < 0.05 vs prestimulus (*n* = 5). cpm: cycles per minute, bpm: beats per minute

To confirm whether LHb activation affects respiratory movement, we stimulated the LHb with 10 to 100 Hz of stimulus frequencies at constant stimulus intensity (300 μA) and 50 to 1000 μA of stimulus intensities at constant stimulus frequency (100 Hz). We then observed the effects on the respiratory movements. In the case of constant current LHb stimulation at 300 μA, 30 Hz or more of stimulus frequencies of stimulation significantly increased the respiratory frequency and (thoracic movement amplitude) × (respiratory frequency) but did not change the thoracic movement amplitude (Fig. 2). Immediately after the LHb stimulation, augmented respiration occurred sometimes. At constant frequency of LHb stimulation at 100 Hz, 300 μA or more of stimulus intensities significantly increased the respiratory frequency and (thoracic amplitude) × (respiratory frequency) (Fig. 3). The thoracic movement amplitude was significantly increased at 700 μA and 1000 μA of stimulus intensities.

To restrict the effect of stimulation to a limited brain region, we considered that 300 μA is the minimal stimulation intensity that can evoke significant respiratory responses and that 100 Hz of stimulation frequency is enough to cause solid and repeatable responses. LHb stimulation at 300 μA and 100 Hz caused a rapid increase in the respiratory frequency and (thoracic movement amplitude) × (respiratory frequency) without changing the thoracic movement amplitude. The LHb stimulation also increased blood pressure, as previously reported [13]. On the other hand, stimulation of the region 1 mm ventrally, rostrally, caudally, and laterally outside the LHb caused little response in respiratory movement or blood pressure (Supplemental Fig. 1).

### Involvement of the monoaminergic system in the respiratory responses to LHb stimulation

Because LHb neurons are known to innervate monoaminergic neurons, we hypothesized that the monoaminergic system mediates LHb-induced respiratory responses. To confirm this, dopaminergic and serotonergic receptor antagonists were administered intravenously.

**Fig. 4:**
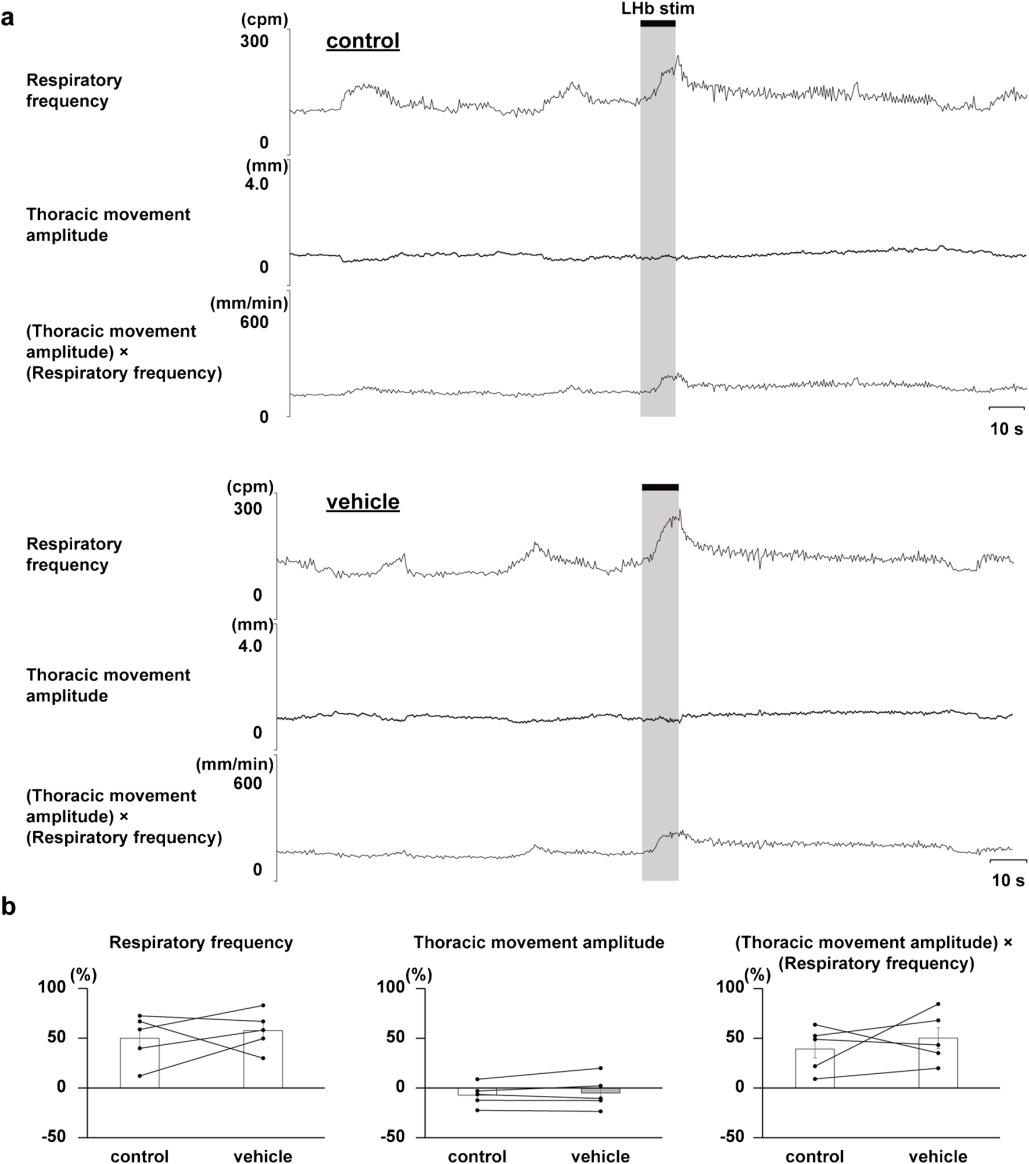
Effects of administering vehicle on LHb-induced respiratory responses. **a** Respiratory responses before (top) and after (bottom) administration of clozapine. The black bars at the top show the period of LHb stimulation. LHb: lateral habenula. cpm: cycles per minute. **b** Changes in respiratory responses after administration of the vehicle compared with respiratory responses before the administration (*n*= 5)

**Fig. 5:**
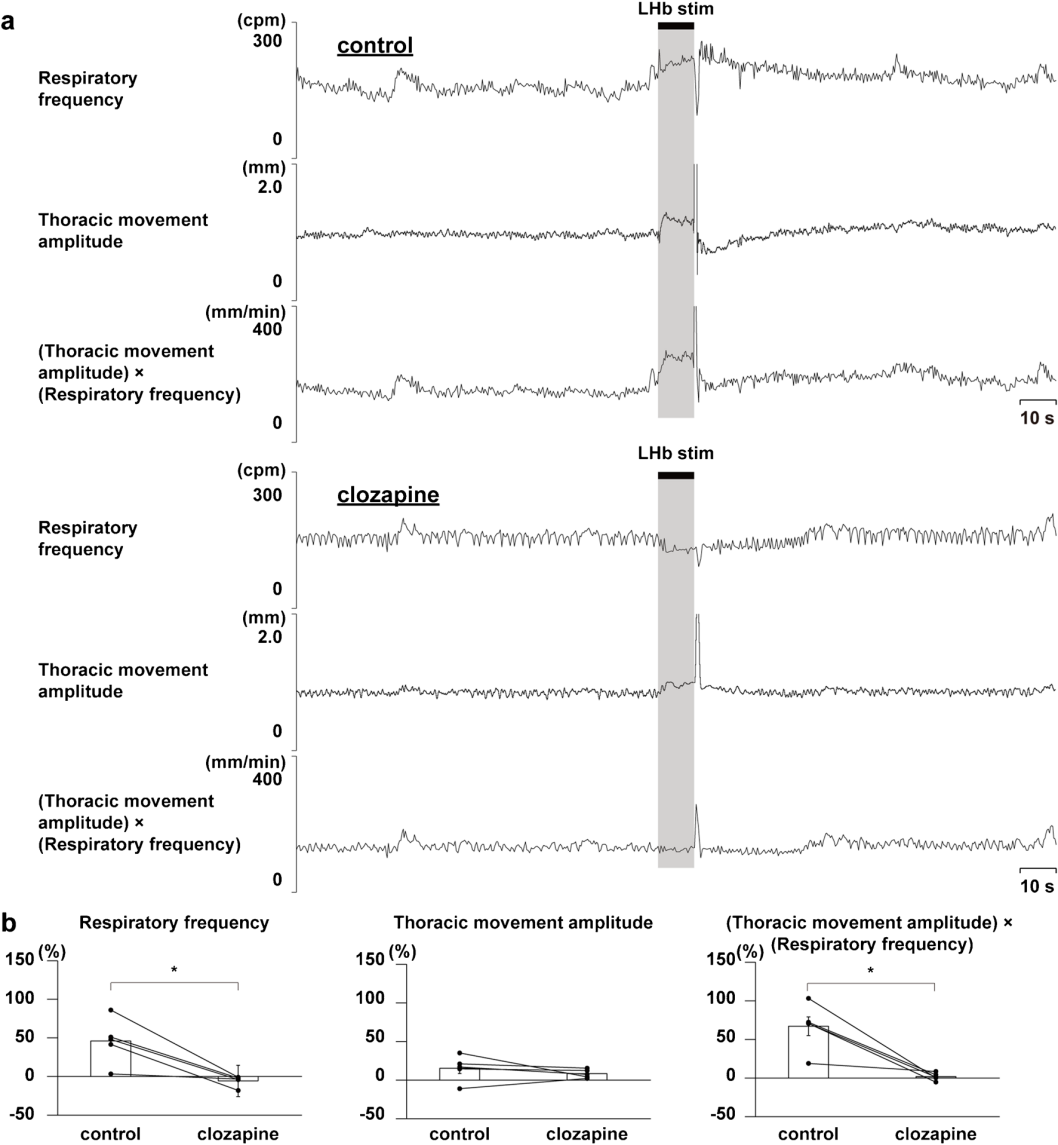
Effects of blockade of dopaminergic receptors on LHb-induced respiratory responses. **a** Respiratory responses before (top) and after (bottom) administration of clozapine. The black bars at the top show the period of LHb stimulation. LHb: lateral habenula. cpm: cycles per minute. **b** Changes in respiratory responses after administration of clozapine compared with respiratory responses before the administration. Asterisks indicate *p* < 0.05 vs prestimulus (*n* = 5)

**Fig. 6:**
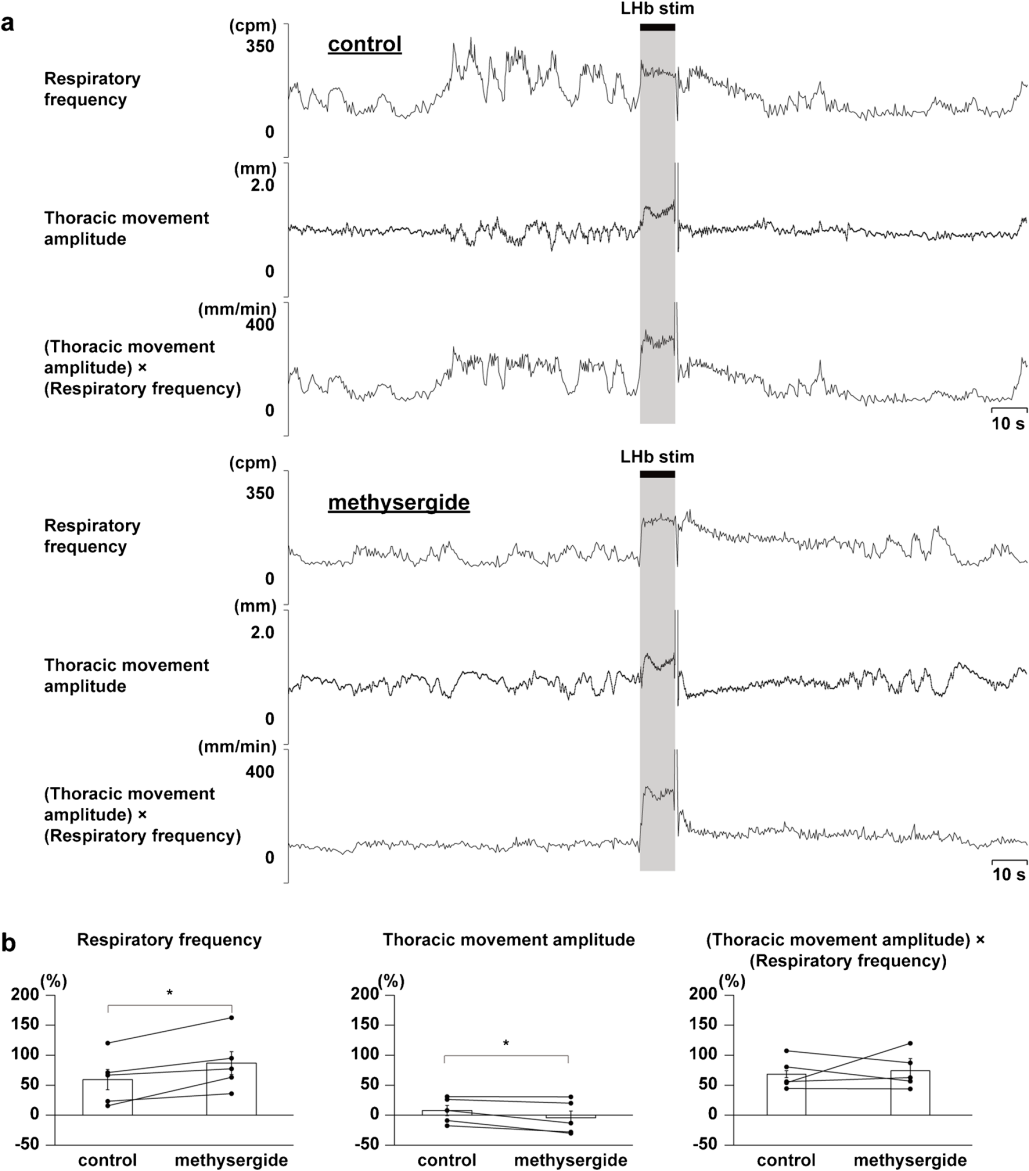
Effects of blockade of serotonergic receptors on LHb-induced respiratory responses. **a** Respiratory responses before (top) and after (bottom) administration of methysergide. The black bars at the top show the period of LHb stimulation. LHb: lateral habenula. cpm: cycles per minute. **b** Changes in respiratory responses after administration of methysergide compared with respiratory responses before the administration. Asterisks indicate *p* < 0.05 vs prestimulus (*n*= 5)

Administration of a dopaminergic receptor antagonist, clozapine, strongly suppressed the LHb-induced respiratory responses, but that of the vehicle did not (Figs. 4 and 5). Administration of a serotonergic receptor antagonist, methysergide, enhanced the LHb-induced increase in respiratory frequency (Fig. 6). Since the thoracic movement amplitude was suppressed during stimulation after the blockade, the (thoracic movement amplitude) × (respiratory frequency) was not changed by the blockade.

### Involvement of VTA dopaminergic neurons in respiratory responses to LHb stimulation

**Fig. 7:**
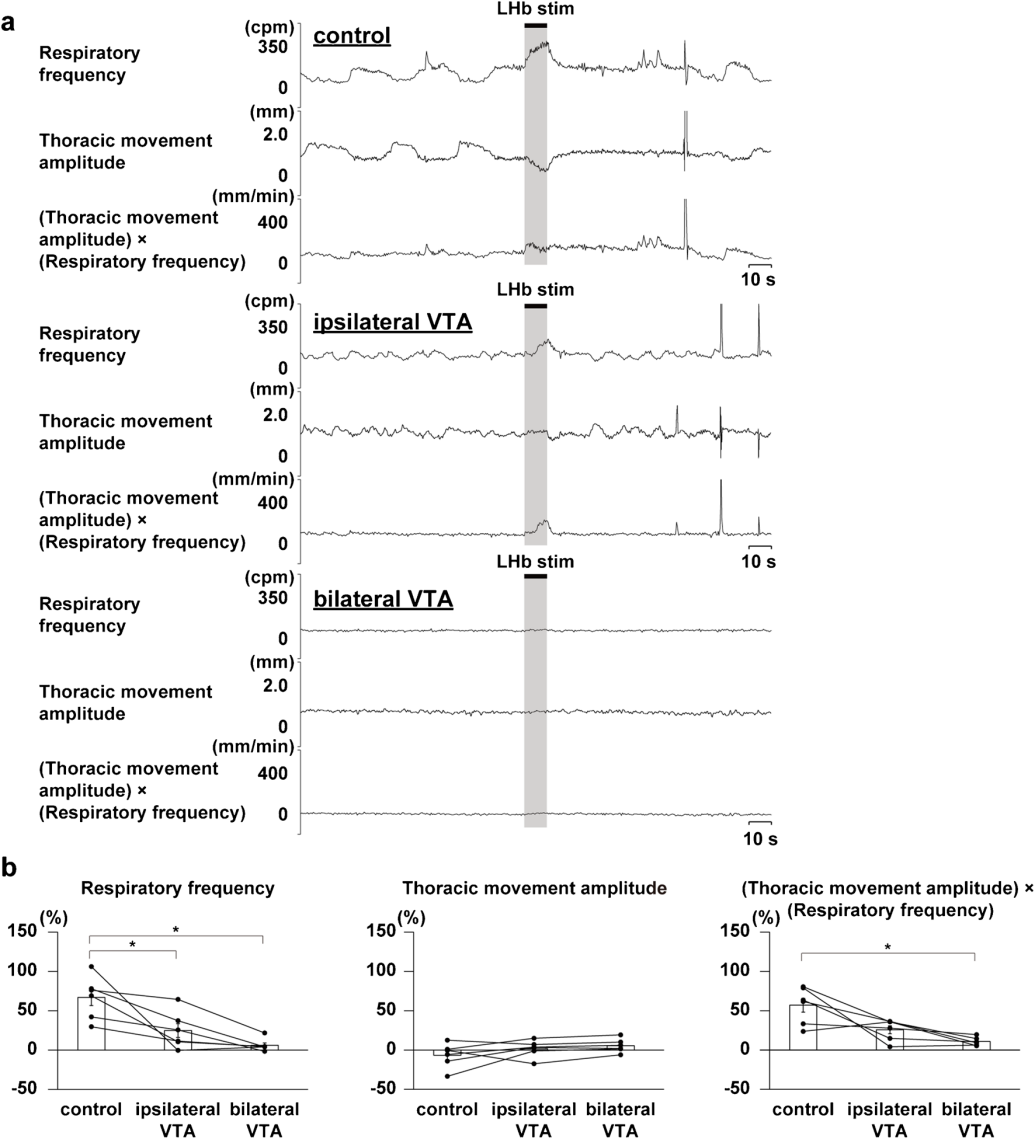
Effects of inactivation of the VTA on LHb-induced respiratory responses. **a** Respiratory responses before (top) and after administration of muscimol to the ipsilateral (middle) and bilateral (bottom) VTA. **b** Changes in respiratory responses after ipsilateral and bilateral administration of muscimol compared with respiratory responses before the administration. Asterisks indicate *p* < 0.05 vs prestimulus (*n*= 6)

Since the LHb-induced respiratory response was almost suppressed during the blockade of dopaminergic receptors, we focused on the dopaminergic system as the mediator of LHb-induced respiratory responses. To elucidate the origin of the dopaminergic system, we investigated the involvement of the VTA, the core of the midbrain dopaminergic system. We locally administered a GABA_A_ receptor agonist, muscimol, to the VTA and examined the effects on the LHb-induced respiratory responses (Fig. 7a). The inactivation of the VTA ipsilateral to the LHb stimulation site remarkably suppressed the LHb-induced increase in respiratory frequency(Fig. 7b). Moreover, the inactivation of the bilateral VTA significantly suppressed the LHb-induced increase in respiratory frequency and (thoracic movement amplitude) × (respiratory frequency).

## Discussion

Here, we investigated the involvement of the LHb in regulation of respiratory movements. Activation of the LHb increased respiratory frequency and minute ventilation, reflected by (thoracic amplitude) × (respiratory frequency). The LHb-induced respiratory responses were observed with stimulation intensity and frequency above a certain level (over 30 Hz and 300 μA). Blockade of dopaminergic receptors strongly suppressed the LHb-induced respiratory responses. On the other hand, blockade of serotonergic receptors enhanced the LHb-induced respiratory responses. Inactivation of the VTA, the center of the dopaminergic system in the midbrain, almost suppressed the LHb-induced respiratory responses. These data indicate that the LHb modulates respiratory movements via the monoaminergic system. The dopaminergic neurons originating from the VTA mainly mediate the LHb-induced respiratory response. Moreover, the serotonergic system suppressively modulates the LHb-induced respiratory responses. The LHb-monoaminergic system is a critical neural circuit regulating stress-related respiratory responses from the LHb.

In this study, to focus on the involvement of the LHb in respiratory regulation, we first confirmed the effects of LHb electrical stimulation on respiratory movements in anesthetized rats because environmental factors also affect respiratory movements in conscious rats. We found that LHb stimulation over 30 Hz and over 300 μA increases respiratory frequency and minute ventilation reflected by (thoracic amplitude) × (respiratory frequency). The response pattern was consistent even with a higher intensity and frequency of LHb stimulation. The LHb neurons respond to aversive events at around 100 Hz [35]. Therefore, to limit the effect of the stimulation in the LHb by evoking sufficient excitation, we determined to use 300 μA, 100 Hz as the electrical stimulation of the LHb for the subsequent experiments. Actually, LHb stimulation with these parameters evoked significant increases in the respiratory frequency, and minute ventilation by stimulation with the same parameters at 1 mm outside the LHb did not affect the respiratory movements. Doan et al. [13] reported that LHb stimulation induces cardiovascular responses in freezing behavior. The stress stimuli typically increase respiratory frequency and do not change or increase the tidal volume of the stimulation [7, 10, 22, 26, 32, 33]. Therefore, the LHb-induced respiratory responses observed in this study might mimic those in stress events, especially in freezing behavior, although additional consideration using conscious rats is needed to observe the LHb-induced respiratory responses with behavioral reactions.

The monoaminergic system, particularly the dopaminergic and serotonergic systems, is involved in the neural network that elicits stress responses. Midbrain dopaminergic neurons show rapid excitation to the aversive stimuli caused by LHb activation [34, 51]. In this study, the systemic blockade of dopamine receptors strongly suppressed the LHb-induced respiratory responses by decreasing changes in respiratory frequency and minute ventilation. This suppression suggests that activation of LHb neurons triggers activation of the respiratory response via the dopaminergic system. In the medullary respiratory center, dopamine increases the respiratory frequency mediated by D_1_ and D_2_ receptors but also decreases it via D_4_ receptors [18, 28]. Therefore, D_1_- and D_2_-mediated pathways may be involved in the respiratory modulation originating from the LHb. Serotonergic neurons in the dorsal raphe are also known to have a relationship with the reward system and to receive inputs from LHb neurons [1]. In this study, blockade of serotonin receptors enhanced the respiratory response to LHb activation. This result suggests that the serotonergic system suppressively modulates the activation of respiration induced by LHb activation. Activating serotonergic inputs to the medullary respiratory center increases and decreases the respiratory frequency. The increase is mediated by 5-HT_2A_ and 5-HT_2C_, whereas the decrease is mediated by 5-HT_1A_ [37]. Therefore, in the LHB-induced respiratory responses, 5HT_1A_-mediated modulation may be a key pathway in the serotonergic system.

The VTA is known to be the center of the midbrain dopaminergic system. VTA neurons respond to stress stimuli [41, 47, 48], have reciprocal projections with LHb neurons [8, 24], and modulate midbrain dopamine neurons [34]. Moreover, dopamine receptors are expressed in the respiratory center in the medulla [18]. In this study, inactivation of VTA neurons by microinjection of a GABA_A_ agonist significantly suppressed the respiratory responses induced by LHb stimulation. This suppression suggests that VTA neurons, probably dopaminergic neurons, mediate the activation of respiratory movements caused by LHb excitation. Thus, dopaminergic neurons in the VTA may be a key mediator for the stress-induced respiratory responses originating from the LHb. Since in this study we inactivated VTA neurons via microinjection of a GABA_A_ agonist, further analysis via optogenetic experiments will help specify the type of VTA neurons related to LHb-induced respiratory responses. Moreover, VTA dopamine neurons receive various types of synaptic inputs, such as glutamatergic, GABAergic, and monoaminergic inputs [24, 38, 44]. To identify the type of inputs to the VTA neurons, observing the effect of blocking these receptors in the VTA on LHb-induced respiratory responses will be helpful.

From an ethical perspective, we used a minimal number of animals in this study. However, we observed consistent and apparent effects of LHb stimulation on respiratory movement. Moreover, blockade of monoaminergic receptors and inactivation of the VTA also consistently and significantly affected the LHb-induced respiratory responses. The usage of anesthetized rats was also helpful in observing consistent results on respiratory movement. Therefore, we concluded that the sample size in this study was sufficient to understand the effects of LHb stimulation on respiratory movement and the monoamine-related mechanisms of LHb-induced respiratory responses.

In conclusion, our results revealed that LHb neurons strongly modulate respiratory movements that are mediated to excitability by dopamine neurons in the VTA and suppressively supported by the serotonergic system. The LHb-monoaminergic pathway, especially the LHb-VTA, may be an essential network for the regulation of respiratory movements during stress events.

## Supplementary Information

Below is the link to the electronic supplementary material.

Supplementary file1 (PDF 148 KB)

## Data Availability

The data that support the findings of this study are available from the corresponding author upon reasonable request.
