# Supplementary material for: The lateral habenula regulates stress-related respiratory responses via the monoaminergic system
Source: Pflugers Arch. 2024 Nov 19;477(3):441–52. doi: 10.1007/s00424-024-03043-7 (PMC11825555; doi:10.1007/s00424-024-03043-7)
Supplement: Supplementary file 1 — Supplementary file1 (PDF 148 KB) [file 424_2024_3043_MOESM1_ESM.pdf]

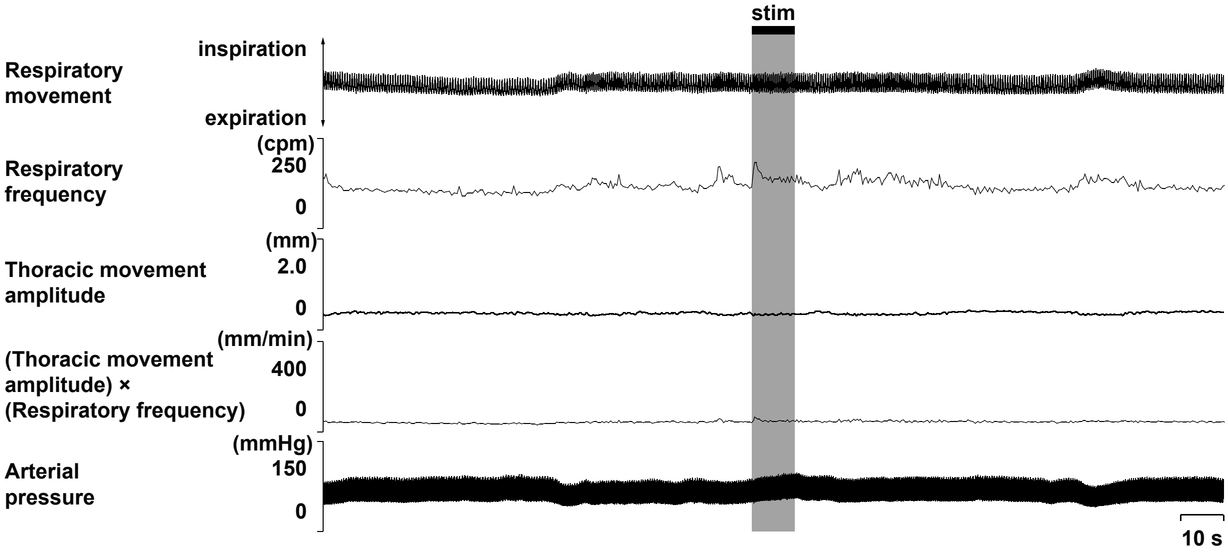


**Supplemental Figure 1**

**Physiological responses to stimulation outside the LHb**

A response during the stimulation (10 s, 300 μA, 100 Hz) to the site 1 mm ventrally from the LHb. The black bar at the top shows the period of stimulation. cpm: cycles per minute.
